# Towards dynamically configured databases for CIFs: the new modulated structures open database at the Bilbao Crystallographic Server

**DOI:** 10.1107/S1600576724007908

**Published:** 2024-09-17

**Authors:** J. Gabirondo-López, I. Gabirondo-López, E. S. Tasci, G. Madariaga

**Affiliations:** a Department of Physics University of the Basque Country UPV/EHU Apartado 644 48080Bilbao Spain; b Department of Physics Engineering, Hacettepe University, Ankara06800, Türkiye; DESY, Hamburg, Germany

**Keywords:** Modulated Structures Open Database, CIF validation, Python web development, Bilbao Crystallographic Server

## Abstract

This article presents a web-based framework to build a database without in-depth programming knowledge given a set of CIF dictionaries and a collection of CIFs. The framework consists of two main elements: the public site that displays the information contained in the CIFs in an ordered manner, and the restricted administrative site which defines how that information is stored, processed and, eventually, displayed. Thus, the web application allows users to easily explore, filter and access the data, download the original CIFs, and visualize the structures via *JSmol*. The modulated structures open database B-IncStrDB, the official International Union of Crystallography repository for this type of material and available through the Bilbao Crystallographic Server, has been re-implemented following the proposed framework.

## Abstract 2

The conceptual exploitation of the CIF scheme allows the design of databases without deep programming knowledge. With this approach, the modulated structures open database B-IncStrDB, the official International Union of Crystallography repository for this type of material already available through the Bilbao Crystallographic Server, has been re-implemented.

## 1. Introduction

The introduction in the early 1990s of crystallographic information files (CIFs) (Hall *et al.*, 1991 ▸), consolidating earlier ideas (Brown, 1983 ▸; Crennell & Brown, 1985 ▸) to standardize the transmission, validation and archiving of crystallographic data, started a new age in the field of structural crystallography. Since then, the number of stored and openly accessible structures with a high degree of quality control has quickly increased. At present, the main goal of the CIF is to constitute the *lingua franca* of all the agents involved in the production, storage and dissemination of (mostly) crystallographic information.

On the other hand, the hierarchy defined by the conceptual sequence (meta-metadata)–(metadata)–(data) expressed by a dictionary definition language (DDL), CIF dictionaries and the CIFs themselves (Hall & McMahon, 2006 ▸) allows the extension from crystallography to other ontologies. The availability of a well defined CIF dictionary language allows easy extension of CIF ontologies to such disparate fields as molecular information (MIF) (Allen *et al.*, 1995 ▸), raw diffraction images (imgCIF) (Bernstein & Hammersley, 2006 ▸) and topology (TopoCIF) (Blatov *et al.*, 2021 ▸), all of which can leverage the availability of dictionary-aware software tools.

From the beginning, the closeness of the structure of a CIF to that of a relational database through the design and organization into categories of the different data names allowed us to think about efficient storage of the information they contained. CIFs were designed to be written, validated and read by programs (while still being human-readable as well). However, and this is a serious drawback, adhering to the standard has generally been a secondary activity of high cost relative to perceived benefit for software developers not directly involved in the validation or exploitation of the information contained in the CIF, despite the tools that already exist to create and manage CIFs [Berman *et al.*, 2006 ▸; International Union of Crystallography (IUCr), since 1993, https://www.iucr.org/resources/cif/software]. This has diminished the reliability of the data contained in the CIFs and contributed to the delay in the migration towards more versatile implementations such as the transition from DDL1 to DDLm dictionaries (Hall & Cook, 2006 ▸; Spadaccini & Hall, 2012 ▸). Consequently, and although fortunately the situation has stabilized over time, many of the existing CIFs are not yet strictly valid [see, for example, Vaitkus *et al.* (2021 ▸)].

It then seems obvious, and more so if we focus on the scope of a database, that validation turns out to be the key process in the usefulness of a CIF for the safeguarding and utility of the data. Said validation can be carried out at several levels: syntactic and data-name validation against a dictionary and data consistency including scientific criteria. Syntactic, semantic (data-name validation against a dictionary) and data consistency (for including scientific criteria) validations are performed by programs such as *VCIF* (McMahon, 1998 ▸, 2006*b* ▸) and *VCIF2* (Todorov & Bernstein, 2008 ▸), *enCIFer* (Allen *et al.*, 2004 ▸), *PyCIFRW* (Hester, 2006 ▸), *publCIF* (Westrip, 2010 ▸), *iotbx.cif* (Gildea *et al.*, 2011 ▸) and, probably the most powerful parser, *COD::CIF::Parser* (Merkys *et al.*, 2016 ▸; Vaitkus *et al.*, 2021 ▸). *PLATON* (Spek, 2003 ▸) (or *checkCIF*; Spek, 2020 ▸) is accepted, by publishers and authors, as the standard tool for the validation of the chemical and crystallographic consistency of single-crystal structural data of small molecules.

A raw validated CIF is a single file consisting of several data blocks (one for each embedded structure) which contain hundreds or thousands of lines, not following a predefined order. In addition, the meaning of the tags or their physical interpretation is not embedded in the file itself. So, although CIFs are human readable in theory, in practice searching for specific data inside a CIF can be a challenging task, particularly for users unfamiliar with, for example, structural crystallography. As the number of published articles is growing exponentially (Fire & Guestrin, 2019 ▸), the only way to handle this huge amount of results and data is to sort them and make them programmatically accessible.

To be scientifically useful, databases recording crystal structures should be not simply collections of CIFs but powerful tools to facilitate data access and management, such as the Protein Data Bank (PDB; Bernstein *et al.*, 1977 ▸), the Crystallography Open Database (COD; Gražulis *et al.*, 2009 ▸), the collection of magnetic structures (MAGNDATA; Gallego *et al.*, 2016*a* ▸,*b* ▸) and many others listed on the website of the IUCr (since 2002, https://www.iucr.org/resources/data/databases). Along with these excellent databases, there coexist less ambitious, but very useful, applications that use CIFs for the exchange of information. Their biggest weakness is usually the use of *adhoc* hard-coded tags: since official dictionaries are revised and expanded over relatively long periods of time, some CIF applications, especially in emerging fields, are built on top of semi-official dictionaries. The subsequent adaptation of these to the finally approved dictionaries may involve a debugging of unknown scope.

In this work, we present a dictionary-independent framework to organize CIF information (although the acronym CIF refers to the crystallographic information framework, in what follows this term will be used as a reference to the syntax of a file, regardless of its content) in databases and make it available via web applications. The main idea is that most of the characteristics of the application (for example, how the stored CIFs are rendered) can be defined by relating them to the tags defined in a set of dictionaries. Thus, the tags are decoupled from the code, so all CIFs are treated equally, and the database can be modified by updating the dictionaries or the CIFs. This allows the maintenance of the database without hard-coding of domain-specific concepts, and it serves as a base database that can be used to store CIFs with different scopes, such as the aforementioned ontologies. Consequently, for this approach to work properly, the correctness of all CIFs contained in the database must be ensured and, therefore, the CIFs shown by the web application are intrinsically valid according to the selected dictionaries. To our knowledge, compared with other databases such as the ones mentioned above, the databases created using this framework have the advantages that they are easily customized, can be used to display the information related to various CIF dictionaries directly and ensure that the CIFs are correct.

The main contributions of this work are the development of a neutral web-based framework, customizable without in-depth programming knowledge, that allows a database to be built with arbitrary information as long as it is supplied in CIF format and there exist dictionaries for its validation. The implementation of this approach, together with its application to the database of modulated structures (B-IncStrDB) included in the Bilbao Crystallographic Server (Aroyo *et al.*, 2006 ▸), is described in the following sections.

## 2. Framework design

The framework proposed in this work allows the creation of web applications to manage and explore databases that gather the information contained in CIFs. The main idea is to define a set of rules that will determine the way CIF information is stored and, eventually, displayed. To do so, the most robust approach is to establish those rules according to the tags defined in a set of CIF dictionaries; thus, all the CIFs that contain the tags defined in those dictionaries will be treated equally. As indicated above, this approach requires that all the CIFs suitable for storage in the database must be valid according to the set of CIF dictionaries, and that all the tags of the files are defined as usable tags within the set of dictionaries. Once both conditions are fulfilled, the CIF is stored, and the information represented in it can be displayed in an appropriate manner.

Such a framework must have two main elements: a database that contains not only the CIF information but also all the information required to display it properly, and a web application, to process all the data contained in the CIFs and to render them in a human-readable way. Consequently, one key element of the web application is CIF validation: to simplify the design of the database application and to ensure both performance and data integrity, it is essential that all ingested data (including CIFs and dictionaries) follow the standards defined by the dictionaries loaded in the application.

To obtain a flexible and easy-to-configure application, the web implementation is divided into two main sites. On the one hand, there is a private administrative site, which is used to manage the information stored in the database, to ensure its correctness and to specify how it is displayed. On the other hand, there is a public site, which renders the information contained in the CIFs of the database. That rendering is done according to the configuration set in the administrative site. The main setup of the web application starts by uploading a set of CIF dictionaries to the administrative site. Once syntactically validated against the DDL on which the dictionary has been built, the tags defined in those dictionaries are stored in the database. Then, those tags that define the meaning, the properties and the category structure of the items included in a CIF can later be used to specify how the CIFs will be stored and rendered. When a CIF is uploaded, it is validated against the set of dictionaries uploaded to the web application, and it is rendered in the public site according to the specifications established in the administrative site.

**Figure 1:**
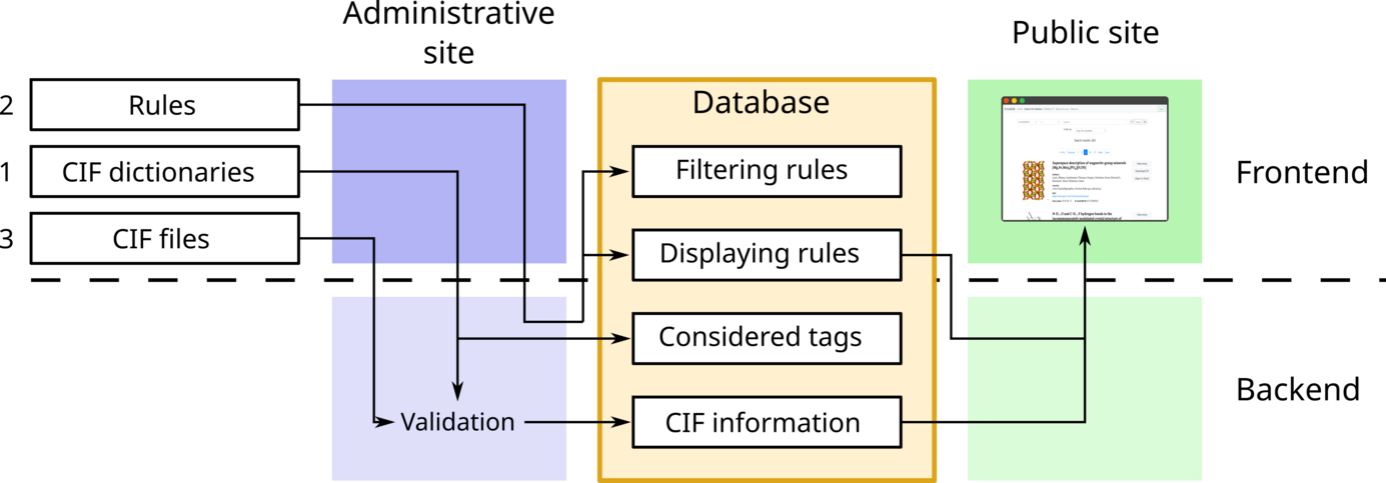
Overall representation of the proposed framework. Administrators provide the CIF dictionaries that will be used for validation, the CIFs and the rules for displaying the information they contain. The backend validates the CIFs against the given dictionaries and stores the information in the database following the rules provided. Finally, users can access the information of the CIFs from the public site.

Each of those sites is divided into two main parts: the backend, which consists of the actual programs that process the requests made by the users and communicates with the database; and the frontend, which contains all the interfaces needed for user interaction. As will be shown in Section 3, the backend is one of the core elements of the framework. It implements all the data processing (including the reading and validation of the CIF dictionaries and files) and the dynamic configuration of the web application. The frontend is responsible for displaying all the information given by the backend. Since it is the part of the framework that is in direct contact with the users, it allows high flexibility in terms of customization: regardless of the backend, the frontend can easily be modified to change the displayed data, the style of the interfaces or any other details needed by the developers. The overall design of the framework can be seen in Fig. 1 ▸: the administrators provide the dictionaries that will be used for validation, some custom rules for processing and displaying the information, and the CIFs to be stored. All this information is stored in the database, so that users can access it by using the public site.

Accordingly, in the next sections (Sections 3 and 4), the administrative site will be explained first, as it contains some essential elements that are required to fully understand the structure of the database, while a general discussion of the public site will be presented in Section 5. Then, a possible frontend is shown in Section 6, which uses the B-IncStrDB as an example, as it is the first crystallographic database built using the proposed framework. Note that the proposed architecture could be implemented by using various platforms or languages, and that the exact tools used in that first application (see Section 6) are only one of the possible options.

## 3. The administrative site

The administrative site allows site managers to specify some parameters of the application related to the visualization of its entries and to the performance of the database. All customizable features can be easily edited and removed, allowing the database to be adapted to any visual requirement or modification in the dictionaries.

Regarding the configuration of the main layout of an entry, the information stored in a record is shown separately in sections. Those sections consist of a title, the position they occupy when rendered and a set of tag categories that are displayed within them. In this context, those categories are the category assignments of each data name in the DDL. Administrators can choose in which order the categories are shown and give a physical meaning to that group by setting a title. Thus, the way in which the information is displayed can be modified to fit the scope of the database.

The web application and the data processing can be dynamically configured by managing the elements that are described in the following subsections.

### 3.1. Dictionaries

The set of dictionaries needed to validate the CIFs define all the tags that could be stored within the database. The specific tags feeding a certain database as well as how it is visually structured are governed by rules established at the administrator level (see Sections 3.3 to 3.7). At present only DDL1 dictionaries are supported.

When a dictionary is uploaded to the database, the first step is to read the file and to check both its syntax and contents (the definitions of the data blocks). If the input dictionary presents any type of error, the upload process is halted and the dictionary will not be stored in the database. Once the correctness of the input dictionary is ensured, the metadata of the file (name of the dictionary, version, last time it was updated…) are retrieved and saved in the database. In the case that the uploaded dictionary is a different version of another dictionary, already in the database, the entry state of all the CIFs that were validated against it is updated, indicating that the CIFs need to be validated again. Finally, the new dictionary is merged with the other dictionaries of the database, so that future validations of CIFs will take into account the restrictions described in this merged file. In this case, that merging procedure is made following the STRICT method described by McMahon (2006*a* ▸) – a fatal error is raised if a data name is multiply defined – but any other merging mode could be used.

### 3.2. CIFs

When a new CIF is uploaded, it is validated against the set of dictionaries of the database. New entries are set as private by default, so they can only be seen by administrators. Each entry receives a unique identifier that can later be used to properly identify that entry. Once a new file is uploaded, its representative figure can be created or uploaded (see Section 6.2.1). All entries can be made public or private at any time, and their figures can also be updated. A deeper explanation of how a CIF is rendered can be found in Section 5.2.

The process of uploading a CIF to the database is very similar to that of the dictionaries. First of all, the CIF is stored in a temporary file for easing the process of reading its contents. The second step is to validate the file against the merged dictionaries stored in the database. If the CIF presents any kind of error or warning, it will not be possible to upload the file. This design decision has been taken in order to ensure that all the CIFs of the database have a minimum base of quality.

After having verified the correctness of the CIF, the next step is to check that all the tags of the file to upload are inside the database. If a tag is not found, the uploading process will be halted and the administrator will be required to update the dictionaries stored in the database and/or upload additional ones. In addition, if the tag appears in the list of excluded tags, this will not be taken into account when performing the validation. With these restrictions, the application ensures that all the tags of the new CIFs are included in the dictionaries stored in the database.

Regarding the storage process of the CIF, first some hard-coded metadata are generated and the tags with their respective values are retrieved. Those metadata are specific for each database: for instance, in the case of the B-IncStrDB, a unique identifier is created, and the values of thetags _publ_section_title, _publ_author_name, _journal_name_full, _journal_year, _journal_volume, _journal_page_first and _journal_page_last are extracted from the CIF and stored in the database. Afterwards, both the metadata and tags are stored in the database. By default, this process also creates a new state for the entry, showing that the entry is private and that it has no errors. Moreover, additional metadata concerning the dictionaries used for validating the CIF are stored in the database. Lastly, the temporary file that is created at the beginning of the process is moved to the database’s directory with an appropriate name, and the original CIF is copied into a common directory for downloading if it is requested by the user. Given that all the curation operations of the database contents are done through CIFs, the correlation between the database contents and the stored CIFs is preserved. The revision histories of the entries are not recorded. Both the database records and the corresponding CIFs are superseded by any updated versions.

### 3.3. Excluded tags

Often, submitted CIFs include information that is outside the scope of the database. For example, CIF generators may include data items with undefined values, data names that belong to dictionaries that are not loaded into the application or information that is not relevant to a first look at the data (for example, a list of reflections). To handle these cases, administrators can define a list of data names that should be ignored during the entry processing. The associated data will not be stored in the database or displayed by the interface. In the case of loops, the whole loop will be skipped if it contains a tag that must be ignored. Thus, it is not required to add all the tags of the loop, but it is sufficient to specify just one of them. It must be emphasized that, as the uploaded CIF remains unchanged, this information is not deleted at all and can be accessed by downloading the original file. (Note that on many occasions the original CIF requires some manual changes to comply with the standard.)

### 3.4. Section structure

The information contained in the CIFs is shown in a layout defined by the administrators. The tags corresponding to certain categories are joined into sections, which means that all the CIFs are displayed in a homogeneous manner that does not depend on the order of the original file. The administrators can create, eliminate and rename those sections, and they can also choose which tag categories are shown inside them.

### 3.5. Tag titles

In order to facilitate the reading of the information shown in the application, especially for inexperienced users, the values of the tags can be accompanied by a more informative title than the actual tag name. Those titles can be set from the administrative site.

### 3.6. Matrices

Some loops are better shown as matrices in order to enhance their readability. Administrators can specify which loops should be shown as matrices by introducing the common root of the tag of the elements of the loop, the dimensions of the matrix and the title that will be shown on the public site.

**Figure 2:**
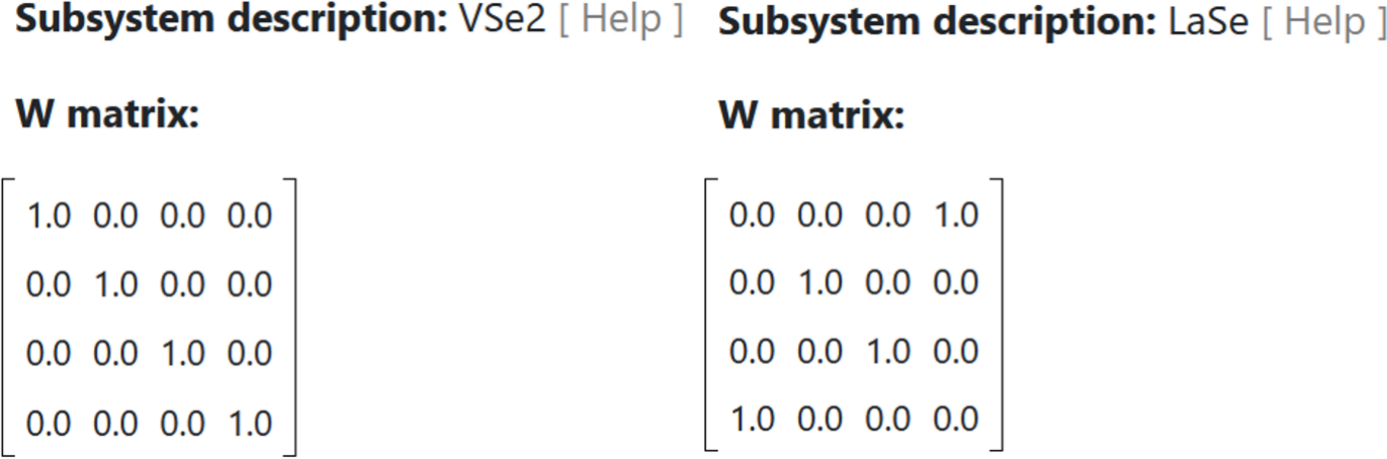
Matrix representation of the values present in the loop example given in the text. For convenience and unlike how they are presented in the database web interface, the matrices have been placed side by side.

For instance, let us consider the case of the tags that are used to represent the cell parameters of each subsystem present in a composite. Those tags are defined in the msCIF dictionary (Madariaga, 2006 ▸). For each subsystem the matrix *W* as defined by van Smaalen (1991 ▸) is represented with a set of tags (whose default values are 0) like _cell_subsystem_matrix_W_i_j, where i and j are integers from 1 to 11, which represent the indices of the element of the matrix. In that case the _cell_subsystem_matrix_W_ string is set as the base of the matrix, as it is the text that is common in all the tags of the elements of the matrix, and the maximum dimensions must be set to 11 and 11. Consequently, for all of the CIFs containing such a loop, it will be shown in its matrix form. A deeper explanation of how a matrix is rendered is given in Section 5.2. An example of such a case can be found in the CIF deposited by Ren *et al.* (1996 ▸):
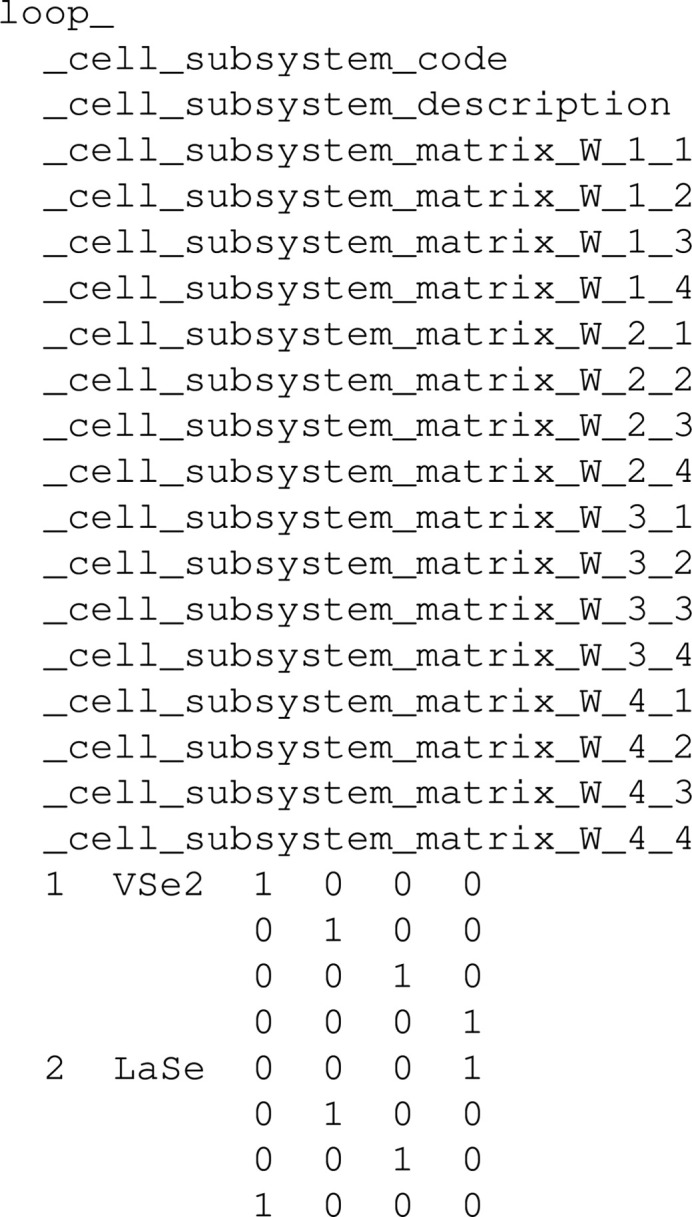


In the public view, the matrices are rendered as shown in Fig. 2 ▸.

### 3.7. Search fields

The search form of the public site is also fully customizable: the tags that will be used to perform the query can be selected through the administrative site. The operator corresponding to a tag is directly selected according to their type. String tags only accept ‘=’ and ‘!=’ operators (equal and not equal, but restricted implicitly to a search by substrings), whereas numerical tags accept ‘=’, ‘!=’, ‘<’, ‘≤’, ‘>’ and ‘≥’, so numerical comparisons can be made between the value of the tag and the input of the search form. Moreover, the selected tags can be shown accompanied by a suitable name instead of the raw tag name, and the order in which they appear can also be edited.

### 3.8. Users

The creation and removal of privileged users or modification of the metadata (first and last names, and the institution) of the existing ones can also be done through the administrative site. This allows dynamic modification of the users that can manage the website. In the future, a group-based organization scheme will be adopted to define the permissions of a set of users.

## 4. Database structure

**Figure 3:**
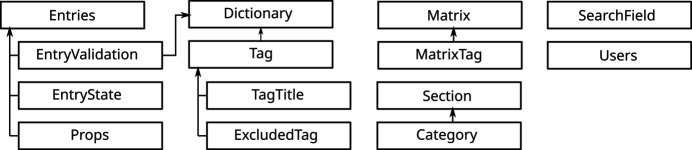
Relations between the main tables that compose the framework. The arrows show how one table relates to another. This distribution of tables has been designed for storing the main aspects of the framework: the information of the CIFs and the dictionaries, the way in which the information is displayed, and the users allowed to manage the database.

The structure of the database (see Fig. 3 ▸) reflects and favors the actions accessed from the administrative site. The tables of the database can be divided into three main groups: the tables containing information on the dictionaries involved in the validation (Dictionary, Tag), the tables containing the entries themselves and their state (Entries, EntryState, EntryValidation and Props), and the tables reflecting the relational aspects between the properties of each entry (Props, Section, Category).

Regarding the dictionaries, it is necessary to keep both their metadata and their contents organized in order to perform CIF validations in an efficient way. In addition, the names of the tags of a dictionary might be difficult to interpret, and therefore they need a human-readable form. Taking these considerations into account, Dictionary stores the metadata of the dictionaries and Tag represents the definition of a tag, indicating the dictionary to which it belongs. Two additional tables (TagTitle and ExcludedTag) are used for keeping track of the human-readable names of the tags and the tags that must not be stored in the database.

Entry management deals with two separate tasks given that it is necessary to keep track of not only the contents of the entry itself but also the metadata of the entry and its state. The table Entries contains all the specific hard-coded CIF metadata. The current state of the entries is stored in the table EntryState. This is very useful for saving several aspects of an entry’s state, for example, whether the entry is publicly available or not, the last time it was revised, or any other aspect required for checking data integrity, given a particular implementation. Database administrators might also find it useful to know the dictionaries that were used for validating an entry. This information is stored in EntryValidation.

The contents (the tags with their respective values) of each specific entry are stored in the table Props. This table stores one row per value in the original CIF, so even though the CIF relational structures are not translated to a series of tables, the proposed architecture allows us to have a single table to store the information contained in all the CIFs of the database. This makes the writing and querying processes easier. Although not mimicking explicitly the table structure of a CIF, the data are structured and stored in the database in terms of tags and values, and the hierarchies and relations are contained and well defined in the dictionaries; therefore, the relational structure of the CIF data can be reconstructed whenever needed. Thus, a singleton occupies a single row of the table, whereas each value inside a loop is represented via a row. To do so, the table has some columns to correctly represent the value (tag name, type and value) and others to refer to the loop (these columns are ignored for singleton values): an identifier of the loop, the position of the tag within that loop and the position of the value in the list of values corresponding to that loop (*i.e.* the position of the row in which the value appears in the loop). As an example, the loop shown below is stored in the Props table as is shown in Table 1 ▸:
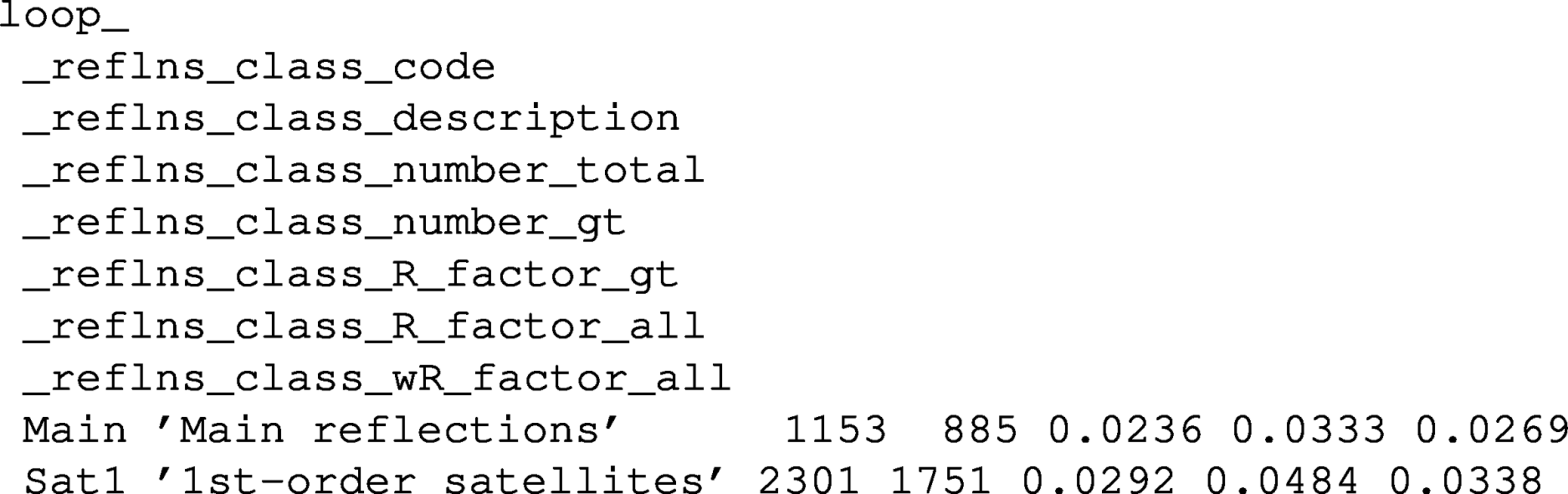


Additional columns are used to store the type of the value, and the raw and processed values of string values that are properly shown in HTML: numerical values are shown along with their standard uncertainty, whereas string values are displayed with unicode characters replacing the special characters defined by the CIF standard.

This design is particularly successful since it avoids the use of fixed-size tables, which force all CIFs to include exactly the same data names. In addition, tags allowed to be looped together inside a CIF may represent a matrix. Therefore, it is convenient to have a table for storing these types of structures. In order to store a matrix, *A*, of dimensions 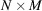 a new row is created in the auxiliary table Matrix and for every matrix element 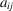 (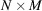 in total) a new entry is created in a table called MatrixTag.

In order to keep the application as human readable as possible, the tags stored in the database can be classified into different categories and, in the same way, categories can be classified into different sections. As these categories and sections may vary depending on the application, it is important to keep them organized in tables. The tables Section and Category are used for storing these two structures. Apart from them, for making complex data queries with ease, the SearchField table keeps the different search operators that can be used when looking for information.

Finally, information about the administrators (name, username, password, email…) is saved in the Users table.

## 5. Public site

The public site of the framework allows users to access the data stored in the database by interacting through the graphical interface. To facilitate the process of looking for data, a search engine that allows complex searches in the database has been designed. Moreover, as has been mentioned before, the public site is highly customizable, allowing easy manipulation of both the search engine and the interfaces for many different types of applications.

### 5.1. The search engine

The search form allows the user to concatenate operations, permitting complex searches and the ability to make a more fine-grained exploration of databases with a considerable number of entries. Although the search engine is based on formal CIF data names, the search forms show an informal name or rubric for each search field. These labels can be modified, with absolute flexibility, from the administrative site.

### 5.2. Rendering the contents of the database

The frontend has been designed to render, in a human-readable way, the information stored in the database. Nevertheless, in order to ensure that the information is displayed correctly, the process of retrieving the data must be efficient and well defined.

First it is necessary to verify that an unprivileged user is not trying to access a private entry file. After that, the application retrieves the metadata of the record selected by the user, which is easily achieved by querying the database with a unique identifier. Once this is done, the application obtains the tags and matrices of the entry. Because of the way in which the database and the interfaces are designed, these two pieces of information require an additional filtering process. Then, the tags of every CIF data block must be classified into the different sections that are defined by the administrators. Hence, the first step is to read the CIF categories that belong to each section, and in the same way the tags that correspond to those CIF categories.

For every tag, its value (together with its standard uncertainty and units, if applicable) and metadata (category of the tag and dictionary where it is defined) are obtained, if it is not part of a loop. On the other hand, the values and metadata of tags belonging to the same loop are stored together using list structures. Finally, as the category of each tag is obtained, the tags are classified into the different sections using those categories.

As far as the matrices are concerned, the first step is to check if the loop identifier indicates it is a matrix. In that case, the values of the tags of the loop are internally represented as separated lists. Then, a reshaping procedure converts these lists into a single matrix. For representing a matrix of dimensions 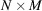, a separate matrix is first created. After that, as every value of the loop is stored in the database with its respective (*i*, *j*) coordinates, each value in the loop is copied into the created matrix. This process is done for every matrix of the input CIF. Finally, the information obtained is rendered.

## 6. A proof of concept: the B-IncStrDB

From the beginning of the Bilbao Crystallographic Server (Aroyo *et al.*, 2006 ▸) and given that there was, within the research group that maintained it, a very active line focused on incommensurate modulated structures, the possibility arose of starting a database dedicated to this novel (in those times) class of materials. Two fundamental milestones contributed to the beginning of this task: the standardization of the description of the modulated structures by the IUCr Commission on Aperiodic Crystals (Chapuis *et al.*, 1997 ▸) and the development of a CIF dictionary including the descriptors proposed by said commission (Madariaga, 2006 ▸). In this way, articles with structural content present in the literature began to be compiled. The information was extracted, analyzed and, so far as was possible, standardized and stored in hand-built CIFs. Obviously, publications grew in number more than it was possible to assimilate. At the same time, the increase in complexity of the analyzed structures required more descriptors than those found in the standard dictionary. Fortunately, the most established refinement program (Petříček *et al.*, 2014 ▸) began producing CIFs automatically. However, the structure of those CIFs varied throughout the program versions, requiring manual adjustments by specialized personnel. In this context the Bilbao Incommensurate Structures Database (B-IncStrDB) was established in 2012. It was implemented on *MySQL* (Widenius *et al.*, 2002 ▸) and was linked to the Bilbao Crystallographic Server, that is, it was publicly accessible. The input interface parsed the CIFs, extracting the information to include in the database. It lacked a validator and only the values of certain hard-coded tags were extracted from the CIF. Taking into account that the authors of the publications sometimes manually modified the CIFs, the task of entering the data could be frustrating. It became evident that there was a need for a CIF validator, not only for the database but for the entire crystallographic server, since it uses CIFs in several of its programs. Even so, some of the initial data storage strategy of the database has been preserved in the current version of the B-IncStrDB. The IUCr has given the database a significant boost by considering it the official repository of structural information for modulated structures and composites, including, in each publication, a direct link to the corresponding entry in the database.

The new version of the modulated structure database (for historical reasons the name B-IncStrDB has been preserved) is a particular implementation of the application described in the previous sections, which uses the official IUCr dictionaries (Hall & McMahon, 2006 ▸) as a reference against which all the entries included in the database have been strictly validated. Currently, two additional local dictionaries are also required to validate some unofficial data names used by structural refinement programs [in practice, only *JANA2020* (Petříček *et al.*, 2023 ▸) and earlier versions are widely used for the refinement of modulated structures] that should disappear and will be properly aliased within the forthcoming DDLm release of the msCIF dictionary (development version available at https://github.com/COMCIFS/Modulated_Structures). The only explicit dependencies on specific data names are the publication data, the chemical formula and, to determine whether the structural data belong to a composite, the number of subsystems it contains. Those items are used as metadata of the entries and shown on the main page of the public site. Even so, the input data themselves constitute the greatest weakness of the database, since sometimes they have had to be manually standardized and could contain errors not originating from the authors of the publication. The most relevant cases are annotated when the structure is displayed. In no case has any value been omitted or corrected, even if it did not have a clear physical meaning.

Regarding the used libraries, the backend is a *Django* (Django Software Foundation, 2019 ▸) application written in Python 3 (Van Rossum & Drake, 2009 ▸). The processing and validation of the CIFs are done by using the code *iotbx.cif* (Gildea *et al.*, 2011 ▸) supplied with the *Computational Crystallography Toolbox* (*cctbx*) (Grosse-Kunstleve *et al.*, 2002 ▸). The present validator is intended to give external users (and also the administrators) a tool to check if their CIFs are appropriate for submission to the database or elsewhere. At the time of writing, the current validator only supports DDL1-based dictionaries. However, the validator is designed to be an interchangeable piece of software that can be updated, completely replaced by a more modern and enhanced program, or even run in a standalone way, separate from the database. This philosophy will allow a rapid transition to DDLm dictionaries. The current list of warnings and errors raised by the validator is shown in Table 2 ▸.

The processed data are stored in a *SQLite* database (Hipp, 2020 ▸). Even though *Django* supports other relational databases such as *MySQL* and *MariaDB*, *SQLite* was chosen in this case because of its easy maintenance and portability. The frontend is mostly based on *Bootstrap* (Twitter, 2019 ▸) and uses *Jinja* to parse the information coming from the backend. It also has some custom-defined CSS rules to modify style-related parameters.

The following sections present an overview of the current status of the database, accessible through the link https://www.cryst.ehu.eus/bincstrdb/.

As the database has been built according to the general design presented in the previous sections, it is divided into two main parts: the public site and the administrative site. Below is a more detailed description of the public site and an explanation of how the administrative site has been adapted for this use case.

### 6.1. The public site

The public site, which tries to be simple in presentation, consists of a search section, the query results and a header, showing a contact link and from where, through a login, the administrative site can be accessed. The validator is accessible, also from the header, regardless of the action being performed on the database.

#### 6.1.1. Visualization of the database contents

**Figure 4:**
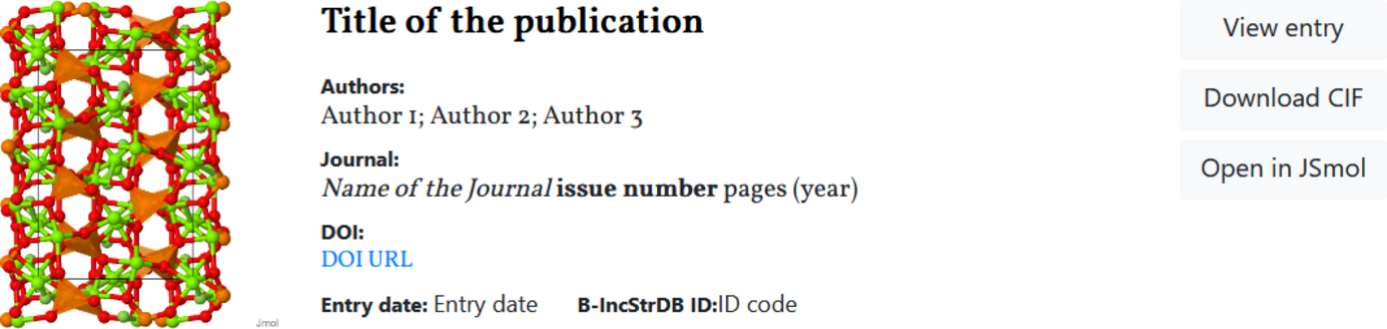
Reduced representation of an entry that shows relevant metadata (article title, authors, journal reference and DOI) and buttons to access the main view of the entry, to download the uploaded CIF or to visualize it via *JSmol*.

**Figure 5:**
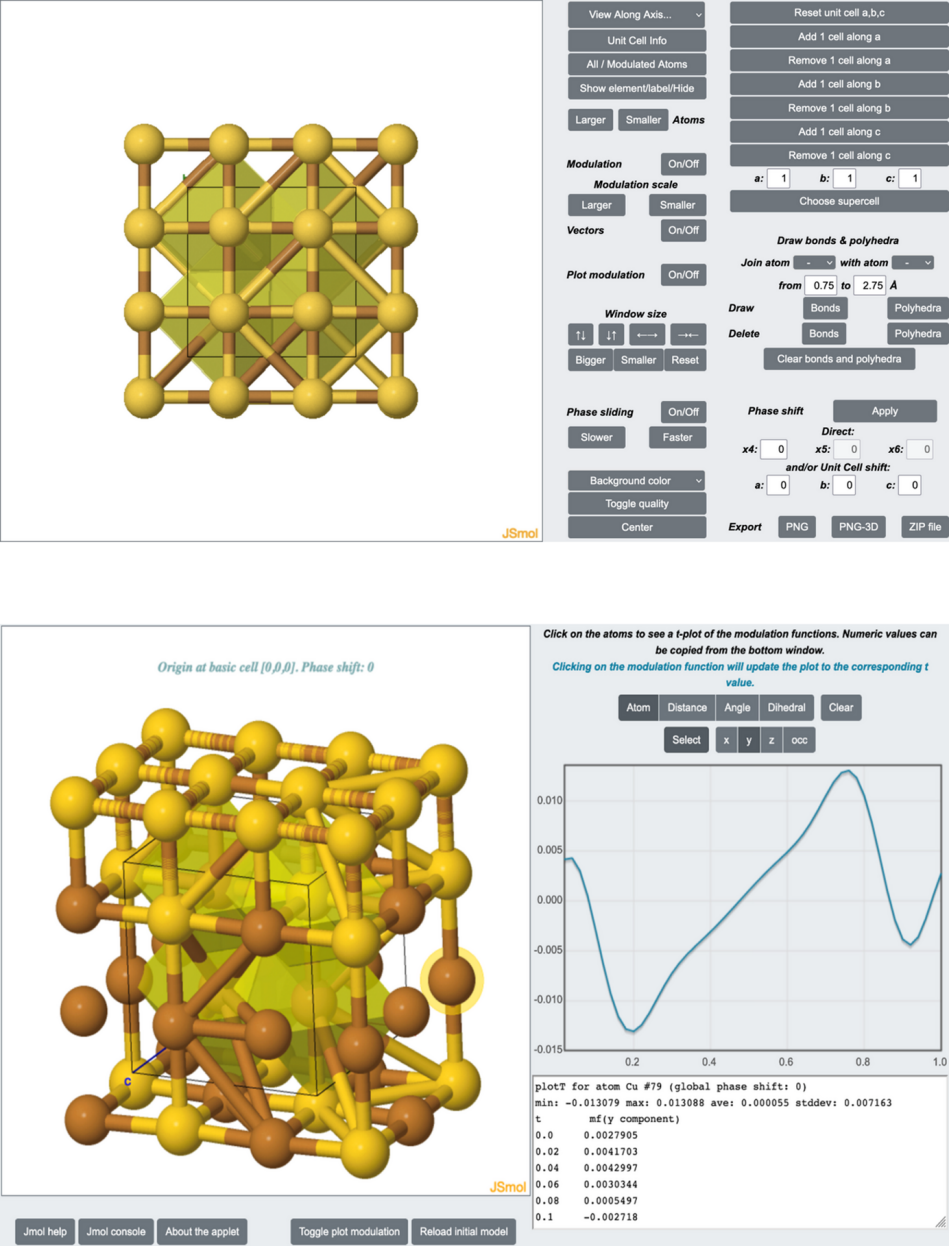
Graphical interface based on *JSmol* (Hanson *et al.*, 2013 ▸). This tool interprets the crystallographic information stored in a CIF and allows its interactive visualization (top), including the atomic modulation functions (bottom) of any selected atom.

The main page of the website shows the entries stored in the database in a paginated list, showing a projection of the structure and the minimum information needed to easily identify the entries (title of the publication, authors, journal reference and DOI) (see Fig. 4 ▸). That information is shown along with three buttons: to view the CIF data; to directly download the uploaded CIF; and to visualize in detail the structure file via *JSmol* (Hanson *et al.*, 2013 ▸) (see Fig. 5 ▸).

When the user clicks on the ‘View entry’ button, the selected information stored in the database is shown in an organized manner. If a CIF contains multiple structures (data blocks), each of them is displayed in a different tab, allowing an easy location of the data associated with each structure. Within each tab, data items are distributed in sections. Each section consists of a title, its position and a set of tag categories that are displayed within it. The description of each item and the data categories included in each displayed section are defined via the administrative site. Thus, the order in which the crystallographic data are displayed is completely determined by the administrators: items will always appear in the same order regardless of the original CIF ordering.

For the user, this style of presentation has advantages over searching within the original CIFs. On the one hand, CIF data names can be replaced by administrator-defined labels, which can be especially helpful for non-expert users. Moreover, where applicable, numerical data are shown explicitly with their respective units, and each item is accompanied by a link that redirects to its definition at the IUCr website. Looped data are presented in two possible ways. By default, CIF loops are shown in tabular form, with each column headed by a user-friendly title or description. However, some of the looped data can also be displayed in matrix form, and this is also chosen on a case-by-case basis by the administrators.

#### 6.1.2. Querying the database

The main page has a search form that allows the user to perform complex queries. The simplest form contains three inputs: the first allows the user to choose one of the tags defined as search tags by the administrators (note that the tag is not shown directly by its name, but by the title defined in the administrative site); using the second the user can select one of the operations corresponding to that tag; and, finally, the third is the text input area. The input area allows the user to use the ‘
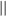
’ operator to perform OR operations: *i.e.* ‘tP6 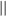 mP12’ means ‘tP6’ OR ‘mP12’.

Apart from simple queries, more complex searches can be done by adding multiple lines to the search form. Each of those form lines works as explained above, and the queries of each line are combined with an AND operator.

#### 6.1.3. Graphical representation of the structures

Great effort has been put into the graphical representation of the specific characteristics of modulated structures. Any click over the thumbnail accompanying each entry brings up a *JSmol*-based page, where each structure contained in the uploaded CIF can be examined, including aspects related to modulation (Fig. 5 ▸).

### 6.2. The administrative site

The administrative site allows modifications online and in real time, with an impact on the information displayed on the public site, which for the user only requires deleting cache data in the browser and reloading the page. Compared with the base framework, the administrative site of B-IncStrDB has been completed with a small application to create the figures that are shown on the public site and another one to add a header to all the CIFs downloaded from it.

#### 6.2.1. Figure generation

As stated before, each entry is accompanied by a figure generated with *JSmol* (Hanson *et al.*, 2013 ▸). Those figures can be created through the administrative site once the CIF is uploaded. An embedded *JSmol* application that loads the CIF can be used to directly create and store a figure that represents the most significant characteristics of the structure. Those thumbnails are saved in the *JSmol*-specific PNGJ format. They are compressed files which include a PNG image, together with the CIF and the state of the structural plot (displayed atoms, bonded atoms, polyhedra if present…). This should allow the user to see, as initial model, the same image as appears in the thumbnail. Moreover, PNGJ files can be uploaded via an input form, so figures can be created outside of the web application using *Jmol* or *JSmol*, and then uploaded to the database.

#### 6.2.2. CIF header

Administrators can also set a multiline string that will be automatically added when a CIF is downloaded from the database. That header can be used to include relevant information: CIF version, the list of dictionaries against which the CIF has been validated and/or from where it was downloaded, for example.

## 7. Conclusions

In the previous sections, a tool has been presented to organize information contained in CIF-like archives in databases, independently of a particular ontology. It only requires the appropriate dictionaries to validate the structure and content of the file. Its design allows great flexibility both to maintain the contents and to vary the type and format of the information presented to users. As an example, the modulated structures database (https://www.cryst.ehu.eus/bincstrdb/, B-IncStrDB) publicly accessible through the Bilbao Crystallographic Server has been presented. At the time of writing this article, the database contains 267 modulated structures (46 of which are composites) that have been, in most cases, manually standardized. All stored CIFs comply with the DDL1 dictionary msCIF.dic. The growth of the database is still far from being completely automated. Although the migration of the dictionary from DDL1 to DDLm will mitigate this situation, at least 100 structures, which have been located in different publications, are waiting as they require a complete CIF; this is because, until not many years ago, information stored in CIF format has been reduced to the average structure. On many occasions, the information about modulation appears only in graphic form. A possible cause is that publishers, beyond the IUCr, do not demand it, perhaps in part because the excellent program *PLATON* (Spek, 2003 ▸) does not yet validate CIFs of modulated structures. The B-IncStrDB could be a good opportunity to centralize the particular information on modulated structures, linked to the rest of the open structural databases that currently exist.

## 8. Future work

The framework presented here allows the creation of highly configurable web applications to exploit CIF-based databases. Even though the current implementation has been successfully used to deploy a referential database such as the B-IncStrDB, the framework needs further development to overcome the challenges posed by the migration from DDL1 to DDLm, and to fit other cases. Firstly, the validator, which is one of the key elements of the framework, must be replaced by another software that deals correctly with DDLm dictionaries and with files conforming to the CIF 2.0 syntax standard. Moreover, the proposed tables are flexible enough to store the new elements introduced by the DDLm language, such as arrays and matrices. As the tag definitions given in the dictionaries are directly loaded into the database, minor modifications of the tables would be required to specify the dictionary version, to include the new high-level metadata and even to manage the aliases correctly. Thus, in principle, it should be even possible to have databases with DDL1 and DDLm dictionaries. Secondly, the framework should be adapted to work with more scalable database platforms such as *MongoDB* and *MySQL*. *Django* is compatible with those technologies, but adopting them would require a major refactor of the code. Finally, in order to promote the creation of open databases, the framework should be made open source so that other developers can deploy their own applications and contribute to the community by improving the current framework. In the short term, we intend to extend the framework for allowing validations against DDLm dictionaries and then open it to the public domain.

## Figures and Tables

**Table 1:** Example of the storage of a loop Note that this table shows a simplified version of the Props table.

Loop order	Tag position	Value position	Tag	Value
1	1	1	_reflns_class_code	Main
1	1	2	_reflns_class_code	Sat1
1	2	1	_reflns_class_description	Main reflections
1	2	2	_reflns_class_description	1st-order satellites
1	3	1	_reflns_class_number_total	1153
1	3	2	_reflns_class_number_total	2301
1	4	1	_reflns_class_number_gt	885
1	4	2	_reflns_class_number_gt	1751
1	5	1	_reflns_class_R_factor_gt	0.0236
1	5	2	_reflns_class_R_factor_gt	0.0292
1	6	1	_reflns_class_R_factor_all	0.0333
1	6	2	_reflns_class_R_factor_all	0.0484
1	7	1	_reflns_class_wR_factor_all	0.0269
1	7	2	_reflns_class_wR_factor_all	0.0338

**Table 2:** List of warnings and errors raised by *iotbx.cif*

Type	Message
Warning	Tag not defined in the dictionary
Warning	Case-sensitive match failure for a value
Warning	Obsolete definition
Error	Value type differs from indicated in data-name definition
Error	Standard uncertainty not applicable to this data name
Error	Value outside of the permitted range
Error	Value not in the enumeration list of the data name
Error	Both data item and exclusive alternate, present in data block
Error	Missing value associated to a data name
Error	Data name cannot be declared in a loop
Error	Data name can only be declared in a loop
Error	Multiple categories mixed in a loop
Error	Value assigned in child data name not found in parent data name
Error	Missing parent data name for loop containing child data name
Error	Missing required reference data name(s) for a loop
